# CNN-based automatic segmentations and radiomics feature reliability on contrast-enhanced ultrasound images for renal tumors

**DOI:** 10.3389/fonc.2023.1166988

**Published:** 2023-06-02

**Authors:** Yin Yang, Fei Chen, Hongmei Liang, Yun Bai, Zhen Wang, Lei Zhao, Sai Ma, Qinghua Niu, Fan Li, Tianwu Xie, Yingyu Cai

**Affiliations:** ^1^Department of Ultrasound, Shanghai General Hospital, Shanghai Jiao Tong University School of Medicine, Shanghai, China; ^2^Department of Pediatrics, Jiahui International Hospital, Shanghai, China; ^3^School of Computer Science and Technology, Taiyuan Normal University, Taiyuan, China; ^4^Institute of Radiation Medicine, Fudan University, Shanghai, China

**Keywords:** automatic segmentation, contrast-enhanced ultrasound, renal tumors, deep learning, UNet

## Abstract

**Objective:**

To investigate the feasibility and efficiency of automatic segmentation of contrast-enhanced ultrasound (CEUS) images in renal tumors by convolutional neural network (CNN) based models and their further application in radiomic analysis.

**Materials and methods:**

From 94 pathologically confirmed renal tumor cases, 3355 CEUS images were extracted and randomly divided into training set (3020 images) and test set (335 images). According to the histological subtypes of renal cell carcinoma, the test set was further split into clear cell renal cell carcinoma (ccRCC) set (225 images), renal angiomyolipoma (AML) set (77 images) and set of other subtypes (33 images). Manual segmentation was the gold standard and serves as ground truth. Seven CNN-based models including DeepLabV3+, UNet, UNet++, UNet3+, SegNet, MultilResUNet and Attention UNet were used for automatic segmentation. Python 3.7.0 and Pyradiomics package 3.0.1 were used for radiomic feature extraction. Performance of all approaches was evaluated by the metrics of mean intersection over union (mIOU), dice similarity coefficient (DSC), precision, and recall. Reliability and reproducibility of radiomics features were evaluated by the Pearson coefficient and the intraclass correlation coefficient (ICC).

**Results:**

All seven CNN-based models achieved good performance with the mIOU, DSC, precision and recall ranging between 81.97%-93.04%, 78.67%-92.70%, 93.92%-97.56%, and 85.29%-95.17%, respectively. The average Pearson coefficients ranged from 0.81 to 0.95, and the average ICCs ranged from 0.77 to 0.92. The UNet++ model showed the best performance with the mIOU, DSC, precision and recall of 93.04%, 92.70%, 97.43% and 95.17%, respectively. For ccRCC, AML and other subtypes, the reliability and reproducibility of radiomic analysis derived from automatically segmented CEUS images were excellent, with the average Pearson coefficients of 0.95, 0.96 and 0.96, and the average ICCs for different subtypes were 0.91, 0.93 and 0.94, respectively.

**Conclusion:**

This retrospective single-center study showed that the CNN-based models had good performance on automatic segmentation of CEUS images for renal tumors, especially the UNet++ model. The radiomics features extracted from automatically segmented CEUS images were feasible and reliable, and further validation by multi-center research is necessary.

## Introduction

Global reports indicate that renal tumors are becoming more prevalent, with over 400,000 new cases and 150,000 deaths annually (1). Renal cell carcinoma (RCC), the most common malignant renal tumor, ranks as the sixth most common cancer among men and the tenth among women, accounting for 5% and 3% of all cancer diagnoses, respectively (2). Accurate diagnosis and prediction of renal malignancies are essential for effective treatment and for further reducing patients’ mortality rates (3). Radiology plays an irreplaceable role in such decision-making. Especially with the development of radiomics, radiology increasingly assists precision medicine and provides accurate medical support. In the past two decades, a new ultrasound technology, contrast-enhanced ultrasound (CEUS), has attracted widespread attention of clinical doctors. For the strictly intravascular nature of ultrasound contrast agents, CEUS is highly sensitive and precise for displaying vessels, even for vessels in tens of micrometers. Significantly, ultrasound contrast agents are safe to use, with no nephrotoxicity and a low incidence of side effects (4). The European Association of Urology (EAU) recommended CEUS for diagnosing renal lesions and identifying the undetermined renal tumors on computerized tomography (CT) or magnetic resonance imaging (MRI) (5–7). Compared with conventional ultrasound, CEUS greatly improves the visibility of microvascular and has a higher signal-to-noise ratio, which has been proved to markedly increase the diagnostic performance for various tumors, and the radiomics studies based on CEUS images are starting to be extensively investigated.

Radiomics involves converting medical image data into a large, quantifiable set of features, which has become a powerful tool for improving accuracy in cancer diagnosis, prognosis, and prediction (8–11). Typically, the radiomics process involves four steps: image acquisition, image segmentation, feature extraction, and data analysis (12). Among those steps, image segmentation is the most crucial and challenging aspect, often attracting the most attention and debate in radiomics analysis (13), since the target area extracted from the entire image could be influenced by many factors. Reliable and reproducible segmentation is therefore essential for data analysis.

Currently, image segmentation in clinical practice is often done manually by radiologists, which is time-consuming, labor-intensive, and subjective. Deep learning techniques, particularly convolutional neural networks (CNNs), have emerged as a promising alternative method (14, 15). In previous studies, CNN-based models have been successfully applied to segmentation of gray-scale ultrasound (US) images of ovarian (15), breast (16), and cervical cancer (17). However, to the best of our knowledge, there has been limited research on CNN-based automatic segmentation of CEUS images for renal tumors. In this study, we aim to evaluate the performance of CNN-based models for automatically segmenting CEUS images of renal tumors and further validate the reliability and reproducibility of extracting radiomics features from automatically segmented target areas.

## Materials and methods

### Patient enrollment

**Figure 1:**
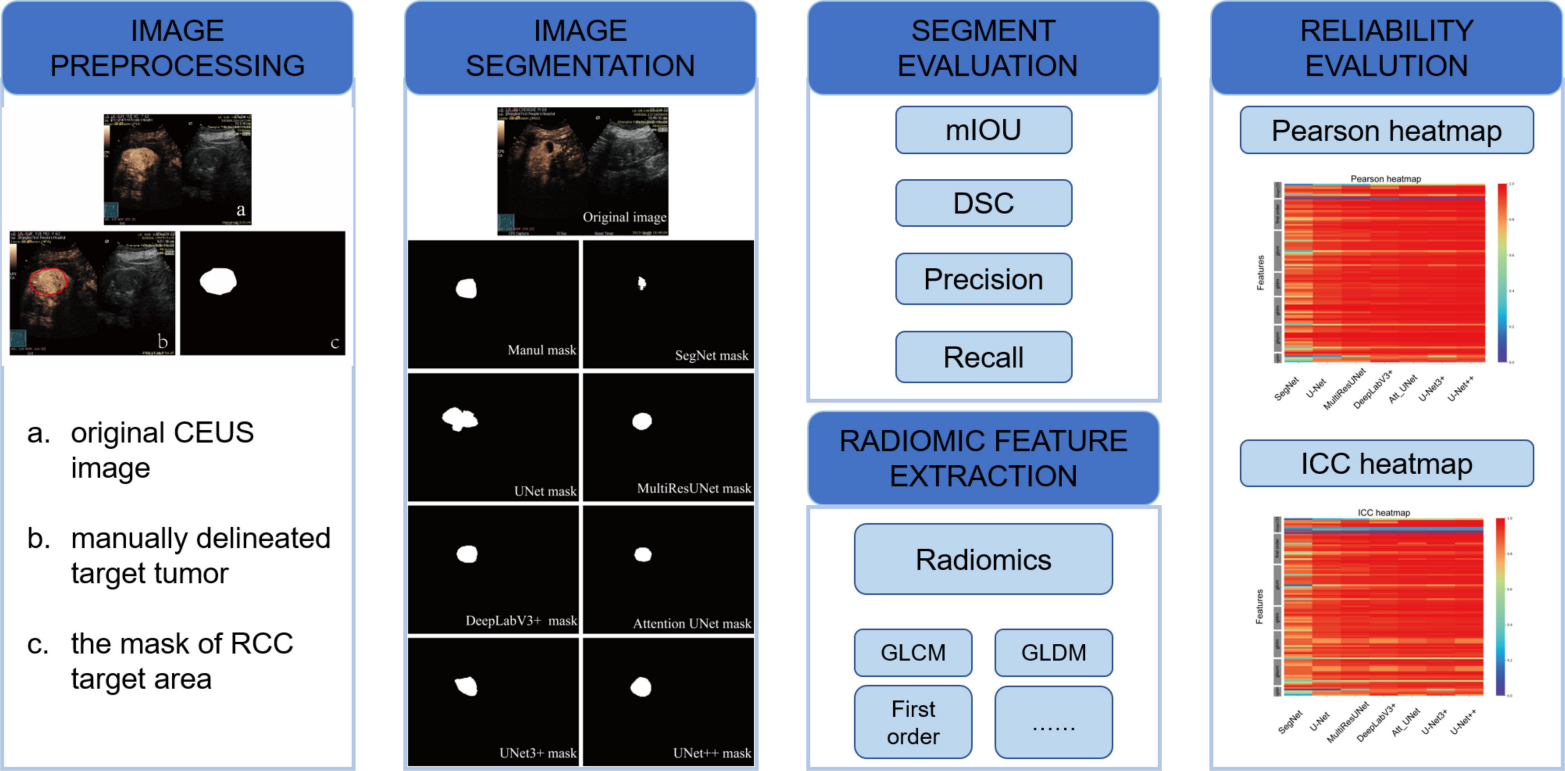
Flowchart of the study with image preprocessing, image segmentation, and evaluation methods.

This is a single-center retrospective study. From January 2013 to December 2016, the imaging data from hospitalized patients with renal tumors were analyzed. Patients were eligible for inclusion if they underwent complete CEUS examination one month before treatment with available pathological data and only one kidney lesion. The study was approved by the hospital ethics committee and all patients provided informed consent and had no side effects from the ultrasound contrast agent. Ninety-four patients were finally enrolled. The data characteristics of the patients are displayed in **Table 1**. The flowchart for the study is shown in **Figure 1**.

**Table 1:** Clinical characteristics of patients with renal tumors in the training and test set.

Category	Patient Characteristics	Images		
		Training Set	Test Set	*P*
**Total Number**	94	3020	335	
**Age (years)**		0.69		
**Average**	60.1	59.4	59.8	
**Range**	27~86	27~86	27~86	
**SD**	12.0	10.9	10.6	
**Gender (%)**		0.70		
**Male**	65(69.1%)	1689(55.9%)	191(57.0%)	
**Female**	29(30.9%)	1331(44.1%)	144(43.0%)	
**Histological Types**		0.12		
**ccRCC**	73(77.7%)	2199(72.8%)	225(67.2%)	
**AML**	13(13.8%)	598(19.8%)	77(23.0%)	
**Others**	8(8.5%)	223(7.4%)	33(9.9%)	

• ccRCC, clear cell renal cell carcinoma; AML, renal angiomyolipoma, others include pRCC, papillary renal cell carcinoma; chRCC, chromophobe renal cell carcinoma and gcRCC, granular cell renal cell carcinoma.

• The p-value is calculated from the univariate association test between different sets; one-factor ANOVA for age, Pearson chi-square for gender and histological types.

### The acquisition and preprocessing of CEUS images

Real-time CEUS examination was performed by senior radiologists *via* LOGIQ E9 (GE Healthcare, USA) and Acuson Sequoia512 (Siemens, USA) with 1.0-5.0 MHz convex probes. SonoVue contrast agent (Bracco, Milan, Italy) that was prepared according to the manufacturer’s recommendations was injected rapidly *via* the right elbow vein (Acuson Sequoia 512 with 1.2 mL and LOGIQ E9 with 2.0 mL). The images started to be continuously recorded for at least 2min and stored in digital imaging.

The RadiAnt DICOM Viewer (Medixant, Poznan, Poland) was used to transform the videos into a series of images before image segmentation, and ideally, 60-80 images were transformed from each video. The images with artifacts caused by respiratory motion and an inadequate field of displaying tumor and surrounding renal parenchyma were excluded. A total of 3355 images extracted from the arterial and venous phases were included in the final analysis. A senior ultrasonic physician manually delineated the region of interest (ROI) of the lesion on each CEUS image using Photoshop (Adobe Photoshop CS6, Adobe Systems Incorporated, USA), and another senior ultrasonic physician confirmed their reliability later. Manually segmented images were used as the ground truth for training and validating CNN-based models.

### Automatic segmentation of CEUS images by different CNN-based models

Generally, a typical CNN architecture includes four types of layers, i.e. convolutional, pooling, fully-connected and non-linearity layers (18). In this study, seven CNN-based models including DeepLabV3+ (19), UNet (20), SegNet (21), MultilResUNet (22), Attention UNet (Att_UNet) (23), UNet3+ (24), UNet++ (25) were used for the automatic segmentation task. To find the best parameters for CNN architectures, we used a comprehensive hyperparameter-tuning procedure. A grid search was used in these procedures to evaluate various combinations of layers and values.

DeepLabV3+, based on the DeepLab system, introduces the Xception model and applied depth-wise separable convolution to both the atrous spatial pyramid pooling and the decoder modules, resulting in more precise object boundary delineation (26). The other six CNN-based models, except for the DeepLabV3+, are all derived from the UNet scheme, but there are some differences between them.

**Figure 2:**
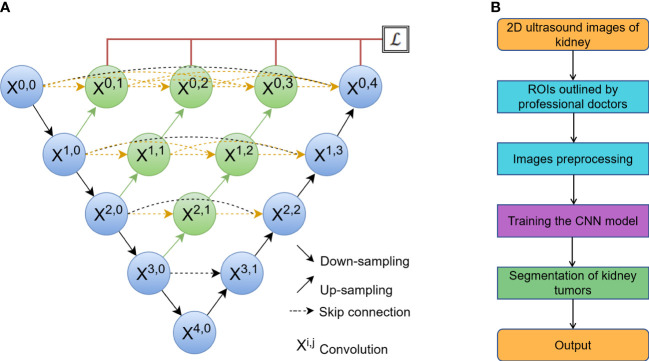
**(A)** The network of a classic U-Net model, where X^i,j^ is the convolution block. The input of each X^i,j^ is concatenated from the up-sampling of X^i+1,j-1^ from the earlier convolution layer of the same dense block and all of X^i,k^ (k< j) from the same pyramid level. **(B)** The process of automatic segmentation by the algorithm.

UNet (**Figure 2A**) has two stages: a down-sampling stage and an up-sampling stage. The left side, known as down-sampling, is an encoder process that uses the max-pooling strategy to compress image features, while the right side, known as up-sampling, is a decoder process that uses the unpooling strategy to output the results. The skip-connection between the encoder and decoder is used to connect the high-dimensional data sets on the left with the low-dimensional data sets on the right to improve the global modeling capability (20).

SegNet is made up of an encoder network, a decoder network, and a pixel-wise classification layer (27). MultiResUNet changes UNet in two ways (22). One is to propose a convolution combination of MultiRes blocks to replace the original two 3 × 3 convolution parts in the model for the varying scales on medical images. Another is to reduce the semantic gap for the skip connection. Att_UNet, which introduces an attention mechanism into UNet, allows the model to highlight key semantic features and dependencies. This makes it easier to find finer details of target objects (28). UNet++ strengthens skip connections by designing an architecture with nested and dense skip connections, aiming to reduce the semantic gap between the encoder and decoder. UNet3+ implements full-scale skip connections as well as deep supervisions to reduce network parameters and improve computation efficiency. The process of automatic segmentation by algorithm was shown in **Figure 2B**.

### Radiomics feature extraction

In order to validate the reliability and reproducibility of automatic segmentations, by using Python 3.7.0 and package PyRadiomics 3.0, the radiomics features were extracted from the automatically segmented and manually segmented target areas. Eighteen first-order statistics and seventy-five texture features were extracted from the gray level co-occurrence matrix (GLCM), the gray level run length matrix (GLRLM), the neighboring gray tone difference matrix (NGTDM), the gray level dependence matrix (GLDM), and the gray level size zone matrix (GLSZM) based on different matrices capturing the spatial intensity distributions at five different scales. Nine shape-based 2D features were extracted from the segmented mask (i.e. the manual or automatic delineated boundary) (29). A total of 102 radiomics features were extracted from each CEUS image for correlation analysis between automatic segmentation and manual segmentation.

### Statistical analysis

The CEUS image dataset was randomly divided into training set and test set for the development of the automatic segmentation models. The following formulae were used to compare each CNN’s performance to ground truth segmentations created by human expert annotators. To define the performance of each CNN-based model in comparison to ground truth segmentations, the following four quantitative indicators were used: mean intersection over union (mIOU), dice similarity coefficient (DSC), precision, and recall, the formulas as follows:


$$Precision=\frac{\text{TP}}{\text{TP}+\text{FP}}$$

$$Recall=\frac{\text{TP}}{\text{TP}+\text{FN}}$$

Pixels or objects are defined into one of four groups, as follows: true positive (TP), false positive (FP), true negative (TN), and false negative (FN). Positive averages the labeled area, whereas negative averages the non-labeled area that can be regarded as background. Precision measures the percentage of accurate predictions. Recall is the same to the model’s TP rate (i.e. the number of TP identified by the model divided by the total number of positives). The mIOU is the average IOU of all classes (e.g. lesion area and non-lesion area for the dataset). The mIOU is:


$$mIOU=\frac{1}{\text{K}+1}\sum_{i=0}^{k}\frac{\text{TP}}{\text{TP}+\text{FP}+\text{FN}}$$

K averages the number of clusters in the filter network. The mIOU measures the overall accuracy (combining elements of precision and recall).


$$DSC=\frac{2\text{TP}}{2\text{TP}+\text{FP}+\text{FN}}$$

With a range of [0,1], DSC measures the overlap between the ground truth and automatic segmentation.

Pearson coefficient and intraclass correlation coefficients (ICC) (two-way mixed effects, single rater, absolute agreement) were used to evaluate the reliability and reproducibility of radiomics features from different automatic segmentation models by comparing them with manual segmentation (17, 29). SPSS 26.0 (IBM, USA) was used to analyze the clinical data. In this work, a *P*<0.05 was regarded as statistical significance.

## Results

### Clinical characteristics of 94 patients with renal tumors

The study included 94 patients with different types of renal tumors, 73 with clear cell renal cell carcinoma (ccRCC), 13 with renal angiomyolipoma (AML), and 8 with other types including 4 with papillary renal cell carcinoma (pRCC), 3 with chromophobe renal cell carcinoma (chRCC) and 1 with granular cell renal cell carcinoma (gcRCC). The average age of the patients was 60.1 years old. The 3355 good CEUS images were finally acquired and divided into training set (3020 images) and test set (335 images) for the development of CNN-based automatic segmentation models. The distribution of age, gender, and histological type was similar between the training and test sets. The clinical characteristics of the patients were listed in **Table 1**.

### Performance of different CNN-based models on automatic segmentation of CEUS images

**Figure 3:**
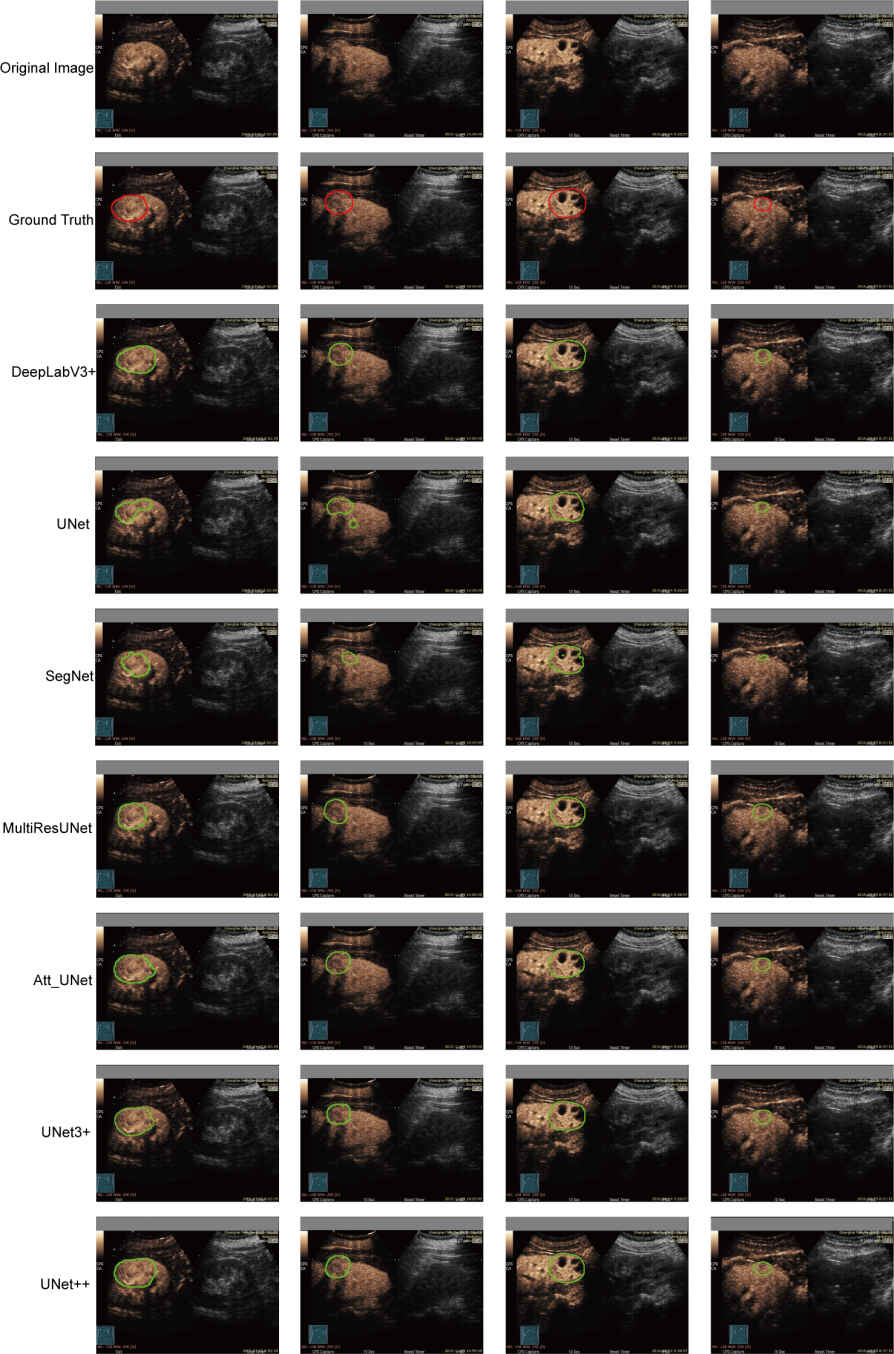
Typical target area segmented by manual delineation, DeepLabV3+, UNet, SegNet, MiltiResUNet, Att_UNet, UNet3+ and UNet++ segmentation models on CEUS images for renal tumors, respectively.

DeepLabV3+, UNet, SegNet, MultilResUNet, Att_UNet, UNet3+, and UNet++ were applied to delineate automatically the target area on CEUS images. **Figure 3** displays typical area segmented by manual delineation, SegNet, UNet, MiltiResUNet, DeepLabV3+, Att_UNet, UNet3+ and UNet++ segmentation models on CEUS images for renal tumors, respectively.

The results of the automatic segmentation models are shown in **Table 2**. The UNet++ model performed the best among all models, with a mIOU value of 93.04%, a DSC value of 92.70%, a precision of 97.43%, and a recall of 95.17%. The SegNet model performed the worst, with mIOU, DSC, precision, and recall values of 81.97%, 78.67%, 93.92%, and 85.29%, respectively. The UNet, MultiResUNet, Att_UNet, and UNet3+ models performed similarly. In terms of DSC, the models were ranked as UNet3++, Att_UNet, MultiResUNet, UNet, and SegNet. All CNN-based models used in the study had a precision greater than 93%, with no recall less than 85%.

**Table 2:** Automatic segmentation accuracy metrics for different CNN-based models.

Method	mIOU	DSC	precision	recall
SegNet	81.97%	78.67%	93.92%	85.29%
UNet	87.79%	86.49%	95.81%	90.70%
MutilResUNet	90.73%	90.06%	95.61%	94.24%
DeepLabV3+	92.09%	91.64%	96.64%	94.84%
Att_UNet	92.70%	92.32%	97.44%	94.79%
UNet3+	92.72%	92.35%	97.56%	93.15%
UNet++	93.04%	92.70%	97.43%	95.17%

### Radiomics features extracted from automatic and manual segmented CEUS images

**Figure 4:**
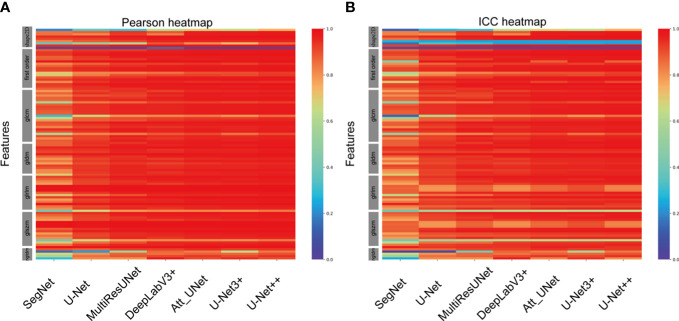
Heat maps of Pearson correlation and intraclass correlation coefficients for radiomics features extracted from CNN-based automatic segmentations.

A total of 102 features were extracted from the segmented ROIs. The majority of the features displayed a strong correlation among the various CNN-based models (**Figure 4A**). Some features, particularly a few shape features, showed weak correlation. In comparison with manual segmentation, the average Pearson coefficients of radiomics features extracted from automatically segmented CEUS images by SegNet, UNet, MultilResUNet, DeepLabV3+, Att_UNet, UNet3+ and UNet++ models were separately 0.81 with 95% confidential interval (CI) of 0.77-0.85, 0.89 (95% CI, 0.87-0.92), 0.92 (95% CI, 0.90-0.95), 0.94 (95% CI, 0.92-0.96), 0.95 (95% CI, 0.93-0.97), 0.94 (95% CI, 0.92-0.96) and 0.95 (95% CI, 0.93-0.97). In **Supplemental Table 1**, the Pearson correlation statistics for all 102 features are shown.

In comparison with manual segmentation, the average ICCs for the assessment of the reproducibility of CEUS image-based radiomics features were 0.77 (95% CI, 0.73-0.81), 0.86 (95% CI, 0.83-0.90), 0.89 (95% CI, 0.86-0.92), 0.90 (95% CI, 0.87-0.93), 0.91 (95% CI, 0.89-0.94), 0.91(95% CI, 0.88-0.94) and 0.92 (95% CI, 0.89-0.95) for SegNet, UNet, MultilResUNet, DeepLabV3+, Att_UNet, UNet3+ and UNet++, respectively (**Figure 4B**). The data of ICCs for all 102 features are presented in **Supplementary Table 2**.

**Figure 5:**
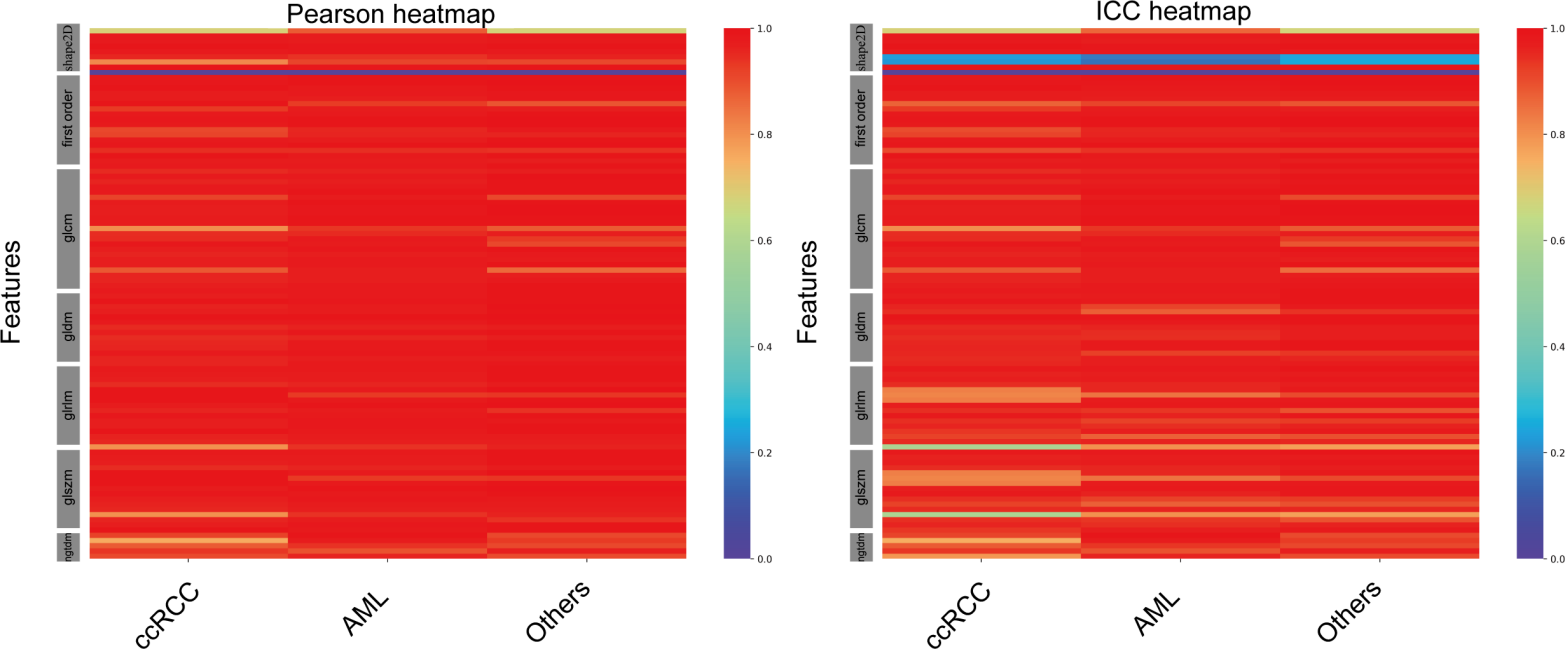
Heat maps of Pearson correlation and intraclass correlation coefficients for radiomics features extracted from manual segmentation and U-Net++ models based on automatic segmentation in different subtypes of renal tumor.

Furthermore, UNet++ achieved the best performance among the seven CNN-based models. Thus, we performed a subgroup analysis to further assess the segmentation effect of the UNet++ model in various pathological categories of renal tumors. According to the types of renal tumors, the test set was split into three sets: CCRCC set (224 images), AML set (77 images), and other tumors set (33 images). The average Pearson coefficient for the assessment of the reliability of CEUS image-based radiomics were 0.95(95%CI, 0.93-0.97), 0.96(95%CI, 0.94-0.98), 0.96(95%CI, 0.94-0.98) for CCRCC, AML and other tumors, respectively. The average ICC for the assessment of the reproducibility of CEUS image-based radiomics features were 0.91 (95%CI, 0.88-0.94), 0.93(95%CI, 0.90-0.96), 0.94(95%CI, 0.91-0.96) for CCRCC, AML and other tumors respectively (**Figure 5**). The data of Pearson coefficients and ICCs for all 102 features in different histological subtypes of renal tumors are presented in **Supplementary Tables 3****,** **4**.

## Discussion

Although CNN-based models have been investigated to automatically segment several types of medical images from patients with various tumor types, few studies have used the technique on the CEUS images for renal tumors. To the best of our knowledge, this is the first study to show the feasibility of utilizing CNN-based models to automatically segment tumor lesions using CEUS images in renal tumor patients. The results suggest that the use of CNN-based models for automatic segmentation on CEUS images of renal tumor is feasible, and the UNet++ model demonstrated demonstrates the best performance. Most of the radiomics features extracted from the automatic segmentation area have good reliability and high repeatability, except for a few shape features.

Manual segmentation is a traditional way of preparing medical images for radiomics analysis, but it is time-consuming and observer-dependent. Using CNN-based models to overcome the shortcomings of manual segmentation is starting to be extensively investigated (15). The deep learning model trained by labeled images can directly process the raw data and standardized the segmented region of interest through the neural network, therefore additional artificial error could be avoided. The use of CNN-based models is highly effective in several imaging modalities, however, it is still challenging for well-known speckle noises, serious cascade, uneven intensity distribution and blurred boundaries in gray-scale ultrasound images (30, 31).

Due to the basic imaging characteristics, gray-scale ultrasonic image is easy to produce speckle noises, so the boundary and texture features are not obvious. Therefore, traditional manual segmentation method is difficult to achieve precise extract results. CEUS imaging operates by detecting the harmonic or subharmonic signals generated from the microbubbles of contrast agents while filtering out the fundamental signal. This allows CEUS images to accurately display microvessels and distinguish between areas with abundant vasculature and those with little to no vasculature, all while maintaining a high signal-to-noise ratio and resolution (32). The development of most malignant tumors relies on neovascularization, thus tumor boundary and inner state could be clearly visualized on CEUS (6, 33). However, there were few studies validating the CNN-based model on automatic segmenting CEUS images for renal tumors. Our study confirmed that seven CNN-based models could achieve a fine DSC ranging from 78.67% to 92.70% for automatically segmenting CEUS images of renal tumors.

A well-proven CNN structure is UNet with superior skip connections design between the encoder and decoder (20). It has been widely used in medical image segmentation and has inspired the development of numerous variations on ultrasound images (15). In this work, UNet, SegNet, MultilResUNet, Att_UNet, UNet3+ and UNet++ were analyzed for their efficacy in automatically segmenting CEUS images of renal tumors. UNet and its variations demonstrated strong performance across all four evaluation metrics. In particular, UNet++ achieved the highest level of performance, with a mIOU of 93.04%, DSC of 92.70%, precision of 97.43% and recall of 95.17%. Powered by redesigned skip connections and deep supervision, UNet++ enables gradual aggregation of the multi-depth image features across the network, which improves the segmentation accuracy of renal tumors (25).

Image segmentation is the second step in radiomics analysis, and the automatically segmented ROI is then used to extract features for further data analysis (12). It has been shown that the image features extracted from both US and CEUS can be used as high-throughput data for clinical outcomes (34). Hence, a stable and reproducible ROI segmentation is crucial for the qualitative and quantitative analysis of medical ultrasound images, as it has a direct impact on follow-up analysis and processing (35). The reproducibility of feature extraction using UNet (ICC: 0.84) and UNet++ (ICC: 0.85) was found to be good in US images for ovarian cancer patients (15). The potential of CEUS-based radiomics for automatic segmentation has yet to be fully explored. In this study, the stability and reproducibility of radiomics features extracted from CEUS images through automatic segmentation were evaluated with average Pearson coefficients ranging from 0.81 to 0.95 and average ICC ranging from 0.77 to 0.92. The results of this study showed that the relationship between automatic and manual segmentation was consistent with the performance of the CNN-based models, meaning that higher DSC values indicated better stability of the radiomics features extracted from the target area through automatic segmentation.

In the evaluation of all CEUS images, the UNet++ model outperformed all other models. The test set was divided into three groups based on the type of renal tumors: ccRCC set (224 images), AML set (77 images), and other subtypes set (33 images). These datasets were then automatically segmented using the UNet++ model. The heatmap results revealed that, among the different subtypes of renal tumors, the correlation between Pearson coefficient (0.96) and ICC (0.93) was stronger for the AML type, as illustrated in **Figure 5**.

There were some limitations in this study. Firstly, there are some discrepancies in the correlation of shape textures, as seen in **Figures 4** and **5**. This may be due to the suboptimal performance of the automatic segmentation algorithms, as demonstrated in **Figure 3**. To enhance the reliability and reproducibility of the delineated areas and radiomics features, further investigations are required to refine the automatic segmentation for CEUS images and enable manual corrections. Secondly, this study did not incorporate the use of the CEUS video format for automatic segmentation, which may result in loss of temporal information. Thirdly, only a limited number of automated techniques were explored, and further examination of other CNN-based models is required. Fourthly, because of the single-center nature of this study, external validation is lacking. Further external validations adding multicenter data are needed to confirm the model.

## Conclusion

Our study demonstrates that automatic segmentation using seven CNN-based models on CEUS images of renal tumors is effective. The UNet++ model is the most efficient algorithm for segmenting CEUS images. The radiomic features extracted from automatically segmented target areas are stable and reproducible.

## Data availability statement

The original contributions presented in the study are included in the article/**Supplementary Material**. Further inquiries can be directed to the corresponding authors.

## Author contributions

YY: Methodology, investigation, analysis data, data curation, visualization, and drafting of the article. FC: Investigation, analysis data, and drafting of the article. HL: Investigation, analysis data. YB: Acquisition and analysis of data. ZW: Visualization, software. LZ: Acquisition and analysis of data. SM: Acquisition and analysis of data. QN: Acquisition and analysis of data. FL: Acquisition, investigation, and funding acquisition. TX: Conceptualization, analysis and interpretation, supervision and writing – review & editing. YC: Conceptualization, analysis and interpretation, supervision, and critical revision of article. The final submitted version of the manuscript was approved by all authors after they thoroughly revised it for important intellectual content. All authors agree to be accountable for the content of the work. All authors contributed to the article and approved the submitted version.

## References

[1] Sung H Ferlay J Siegel RL Laversanne M Soerjomataram I Jemal A. Global cancer statistics 2020: GLOBOCAN estimates of incidence and mortality worldwide for 36 cancers in 185 countries. CA Cancer J Clin (2021) 71(3):209–49. doi: 10.3322/caac.21660 33538338

[2] Capitanio U Bensalah K Bex A Boorjian SA Bray F Coleman J. Epidemiology of renal cell carcinoma. Eur Urol (2019) 75(1):74–84. doi: 10.1016/j.eururo.2018.08.036 30243799 PMC8397918

[3] Shaw G. The silent disease. Nature (2016) 537(7620):S98–9. doi: 10.1038/537S98a 27626783

[4] Cantisani V Bertolotto M Clevert DA Correas JM Drudi FM Fischer T. EFSUMB 2020 proposal for a contrast-enhanced ultrasound-adapted bosniak cyst categorization - position statement. Ultraschall Med (2021) 42(2):154–66. doi: 10.1055/a-1300-1727 33307594

[5] Ljungberg B Albiges L Abu-Ghanem Y Bensalah K Dabestani S Fernández-Pello S. European Association of urology guidelines on renal cell carcinoma: the 2019 update. Eur Urol (2019) 75(5):799–810. doi: 10.1016/j.eururo.2019.02.011 30803729

[6] Furrer MA Spycher SCJ Büttiker SM Gross T Bosshard P Thalmann GN. Comparison of the diagnostic performance of contrast-enhanced ultrasound with that of contrast-enhanced computed tomography and contrast-enhanced magnetic resonance imaging in the evaluation of renal masses: a systematic review and meta-analysis. Eur Urol Oncol (2020) 3(4):464–73. doi: 10.1016/j.euo.2019.08.013 31570270

[7] Zhu J Li N Zhao P Wang Y Song Q Song L. Contrast-enhanced ultrasound (CEUS) of benign and malignant renal tumors: distinguishing CEUS features differ with tumor size. Cancer Med (2022) 12(3):2551–9. doi: 10.1002/cam4.5101 PMC9939203 36057970

[8] Zhang L Sun K Shi L Qiu J Wang X Wang S. Ultrasound image-based deep features and radiomics for the discrimination of small fat-poor angiomyolipoma and small renal cell carcinoma. Ultrasound In Med Biol (2023) 49(2):560–68. doi: 10.1016/j.ultrasmedbio.2022.10.009 36376157

[9] Habibollahi P Sultan LR Bialo D Nazif A Faizi NA Sehgal CM. Hyperechoic renal masses: differentiation of angiomyolipomas from renal cell carcinomas using tumor size and ultrasound radiomics. Ultrasound In Med Biol (2022) 48(5):887–94. doi: 10.1016/j.ultrasmedbio.2022.01.011 35219511

[10] Zhang H Huo F. Prediction of early recurrence of HCC after hepatectomy by contrast-enhanced ultrasound-based deep learning radiomics. Front In Oncol (2022) 12:930458. doi: 10.3389/fonc.2022.930458 PMC9554932 36248986

[11] Lambin P Leijenaar RTH Deist TM Peerlings J de Jong EEC van Timmeren J. Radiomics: the bridge between medical imaging and personalized medicine. Nat Rev Clin Oncol (2017) 14(12):749–62. doi: 10.1038/nrclinonc.2017.141 28975929

[12] Lambin P Rios-Velazquez E Leijenaar R Carvalho S van Stiphout RGPM Granton P. Radiomics: extracting more information from medical images using advanced feature analysis. Eur J Cancer (2012) 48(4):441–46. doi: 10.1016/j.ejca.2011.11.036 PMC4533986 22257792

[13] Gillies RJ Kinahan PE Hricak H. Radiomics: images are more than pictures, they are data. Radiology (2016) 278(2):563–77. doi: 10.1148/radiol.2015151169 PMC4734157 26579733

[14] van Timmeren JE Cester D Tanadini-Lang S Alkadhi H Baessler B. Radiomics in medical imaging-"how-to" guide and critical reflection. Insights Imaging (2020) 11(1):91. doi: 10.1186/s13244-020-00887-2 32785796 PMC7423816

[15] Jin J Zhu H Zhang J Ai Y Zhang J Teng Y. Multiple U-Net-Based automatic segmentations and radiomics feature stability on ultrasound images for patients with ovarian cancer. Front In Oncol (2020) 10:614201. doi: 10.1016/j.neunet.2019.08.025 PMC7930567 33680934

[16] Yap MH Goyal M Osman FM Martí R Denton E Juette A. Breast ultrasound lesions recognition: end-to-end deep learning approaches. J Med Imaging (Bellingham) (2019) 6(1):11007. doi: 10.1117/1.JMI.6.1.011007 PMC6177528 30310824

[17] Jin J Zhu H Teng Y Ai Y Xie C Jin X. The accuracy and radiomics feature effects of multiple U-net-Based automatic segmentation models for transvaginal ultrasound images of cervical cancer. J Digit Imaging (2022) 35(4):983–92. doi: 10.1007/s10278-022-00620-z PMC9485324 35355160

[18] LeCun Y Bengio Y Hinton G. Deep learning. Nature (2015) 521(7553):436–44. doi: 10.1038/nature14539 26017442

[19] da Cruz LB Júnior DAD Diniz JOB Silva AC de Almeida JDS de Paiva AC. Kidney tumor segmentation from computed tomography images using DeepLabV3+ 2.5D model. Expert Syst Appl (2022) 192:116270. doi: 10.1016/j.eswa.2021.116270

[20] Ronneberger O Fischer P Brox T. U-Net: convolutional networks for biomedical image segmentation. (2015) 9351:234–41. doi: 10.1007/978-3-319-24574-4_28

[21] Alqazzaz S Sun X Yang X Nokes L. Automated brain tumor segmentation on multi-modal MR image using SegNet. Comput Visual Media (2019) 5(2):209–19. doi: 10.1007/s41095-019-0139-y

[22] Ibtehaz N Rahman MS. MultiResUNet: rethinking the U-net architecture for multimodal biomedical image segmentation. Neural Networks (2020) 121:74–87. doi: 10.1016/j.neunet.2019.08.025 31536901

[23] Mishra D Chaudhury S Sarkar M Soin AS. Ultrasound image segmentation: a deeply supervised network with attention to boundaries. IEEE Trans BioMed Eng (2019) 66(6):1637–48. doi: 10.1109/TBME.2018.2877577 30346279

[24] Huang H Lanfen L Ruofeng T Hongjie H Qiaowei Z Yutaro I. (2020). UNet 3+: a full-scale connected UNet for medical image segmentation. arXiv 2004.08790

[25] Zhou Z Siddiquee MMR Tajbakhsh N Liang J. UNet++: redesigning skip connections to exploit multiscale features in image segmentation. IEEE Trans Med Imaging (2020) 39(6):1856–67. doi: 10.1109/tmi.2019.2959609 PMC7357299 31841402

[26] Chen L-C Zhu Y Papandreou G Schroff F Adam H. Encoder-decoder with atrous separable convolution for semantic image segmentation. computer vision – ECCV Vol. 2018. . Cham: Springer International Publishing (2018). doi: 10.1007/978-3-030-01234-2_49

[27] Badrinarayanan V Kendall A Cipolla R. SegNet: a deep convolutional encoder-decoder architecture for image segmentation. IEEE Trans Pattern Anal Mach Intell (2017) 39(12):2481–95. doi: 10.1109/TPAMI.2016.2644615 28060704

[28] Pal D Reddy PB Roy S. Attention UW-net: a fully connected model for automatic segmentation and annotation of chest X-ray. Comput Biol Med (2022) 150:106083. doi: 10.1016/j.compbiomed.2022.106083 36137316

[29] Zwanenburg A Vallières M Abdalah MA Aerts HJWL Andrearczyk V Apte A. The image biomarker standardization initiative: standardized quantitative radiomics for high-throughput image-based phenotyping. Radiology (2020) 295(2):328–38. doi: 10.1148/radiol.2020191145 PMC7193906 32154773

[30] Chen G Yin J Dai Y Zhang J Yin X Cui L. A novel convolutional neural network for kidney ultrasound images segmentation. Comput Methods Programs BioMed (2022) 218:106712. doi: 10.1016/j.cmpb.2022.106712 35248816

[31] Hsiao C-H Sun T-L Lin P-C Peng T-Y Chen Y-H Cheng C-Y. A deep learning-based precision volume calculation approach for kidney and tumor segmentation on computed tomography images. Comput Methods Programs BioMed (2022) 221:106861. doi: 10.1016/j.cmpb.2022.106861 35588664

[32] Averkiou MA Bruce MF Powers JE Sheeran PS Burns PN. Imaging methods for ultrasound contrast agents. Ultrasound Med Biol (2020) 46(3):498–517. doi: 10.1016/j.ultrasmedbio.2019.11.004 31813583

[33] Forsberg F Kuruvilla B Pascua MB Chaudhari MH Merton DA Palazzo JP. Comparing contrast-enhanced color flow imaging and pathological measures of breast lesion vascularity. Ultrasound In Med Biol (2008) 34(9):1365–72. doi: 10.1016/j.ultrasmedbio.2008.02.010 PMC2556965 18436369

[34] Guo SY Zhou P Zhang Y Jiang LQ Zhao YF. Exploring the value of radiomics features based on b-mode and contrast-enhanced ultrasound in discriminating the nature of thyroid nodules. Front In Oncol (2021) 11:738909. doi: 10.3389/fonc.2021.738909 PMC8551634 34722288

[35] Hatt M Lee JA Schmidtlein CR Naqa IE Caldwell C De Bernardi E. Classification and evaluation strategies of auto-segmentation approaches for PET: report of AAPM task group no. 211. Med Phys (2017) 44(6):e1-e42. doi: 10.1002/mp.12124 28120467 PMC5902038

